# Room-temperature serial synchrotron crystallography structure of *Spinacia oleracea* RuBisCO

**DOI:** 10.1107/S2053230X24004643

**Published:** 2024-05-29

**Authors:** Monika Bjelčić, Oskar Aurelius, Jie Nan, Richard Neutze, Thomas Ursby

**Affiliations:** a MAX IV Laboratory, Lund University PO Box 118 221 00Lund Sweden; b Department of Chemistry and Molecular Biology University of Gothenburg Medicinaregatan 9C 413 90Gothenburg Sweden; University of Essex, United Kingdom

**Keywords:** RuBisCO, SSX, serial synchrotron crystallography, room-temperature crystallography, spinach, *Spinacia oleracea*

## Abstract

Ribulose-1,5-bisphosphate carboxylase/oxygenase (RuBisCO) is the enzyme responsible for the first step of carbon dioxide (CO_2_) fixation in plants, which proceeds via the carboxylation of ribulose 1,5-biphosphate. Because of the enormous importance of this reaction in agriculture and the environment, there is considerable interest in the mechanism of fixation of CO_2_ by RuBisCO. Here, a serial synchrotron crystallography structure of spinach RuBisCO is reported at 2.3 Å resolution. This structure is consistent with earlier single-crystal X-ray structures of this enzyme and the results are a good starting point for a further push towards time-resolved serial synchrotron crystallography in order to better understand the mechanism of the reaction.

## Abstract 2

The first room-temperature serial crystallography structure of RuBisCO is presented.

## 1. Introduction

Photosynthesis provides the principal source of energy input into the biosphere. In plants and many photosynthetic bacteria, reactions complementary to photosynthesis lead to the fixation of carbon dioxide (CO_2_) from the atmosphere and water into sugar in the absence of light (Calvin & Benson, 1948 ▸; Bassham *et al.*, 1950 ▸). Ribulose-1,5-bisphosphate carboxylase/oxygenase (RuBisCO) catalyses the addition of CO_2_ to ribulose 1,5-biphosphate (RuBP), using magnesium as a cofactor, once the enzyme has been activated by the addition of CO_2_ to a conserved active-site lysine residue to form a carbamate. For every three RuBP molecules consumed by this carboxylation reaction, six molecules of 3-phosphoglycerate (3-PGA) result, of which five are utilized in the Calvin cycle to regenerate RuBP and the sixth is ultimately converted into starch. Through this mechanism, RuBisCO is responsible for the annual fixation of approximately 10^11^ tons of CO_2_ from the atmosphere and its function is implicit in any strategy to mitigate the effects of global warming. RuBisCO also catalyses the first step in photorespiration, the oxygenation of RuBP, which acts in competition to the CO_2_-fixation reaction and lowers the overall enzymatic efficiency. RuBisCO is the most expressed enzyme in plant leaves, with estimates that 18.2 ± 6.2% of leaf-protein nitrogen is used in RuBisCO (Luo *et al.*, 2021 ▸), making it the most abundant protein on earth. RuBisCO is an ancient enzyme and its rate of catalysis during carbon fixation is very slow, with only three to ten CO_2_ molecules added per second (Ellis, 2010 ▸). Considerable efforts have therefore been made to improve the rate of turnover of this enzyme and thereby potentially improve upon the overall efficiency of CO_2_ fixation (Liu *et al.*, 2010 ▸; Ellis, 2010 ▸).

Crystal structures of RuBisCO from *Rhodospirillum rubrum* (Schneider *et al.*, 1986 ▸, 1990 ▸), tobacco (Curmi *et al.*, 1992 ▸; Schreuder *et al.*, 1993 ▸), spinach (Andersson *et al.*, 1989 ▸) and the green alga *Chlamydomonas reinhardtii* (Taylor *et al.*, 2001 ▸) were solved by X-ray crystallography several decades ago. In plants and cyanobacteria, RuBisCO forms a complex hexadecameric oligomer consisting of eight copies of the large (RbcL) subunit and eight copies of the small (RbcS) subunit. The active site of the enzyme is located at the interface between two RbcL subunits and contains a high concentration of conserved positively and negatively charged residues within a 10 Å radius of the catalytic residue (Andersson *et al.*, 1989 ▸). Lys201L is activated by the addition of CO_2_ and the labile carbamate form of this residue coordinates an Mg^2+^ ion that participates in catalytic activity. X-ray structures of spinach RuBisCO (SpRub) have been solved at resolutions as high as 1.6 Å (Andersson, 1996 ▸) and have revealed the enzyme in its activated form, in complex with its natural substrate RuBP (Taylor & Andersson, 1997*b* ▸) and in complex with the reaction product 3-PGA (Taylor & Andersson, 1997*a* ▸), as well as in complexes with other known inhibitors (Taylor *et al.*, 1996 ▸). These structures have yielded a picture of the structural mechanism of enzyme catalysis, but many details remain elusive (Andersson & Taylor, 2003 ▸).

Time-resolved crystallography aims to reveal structural details of enzyme catalysis in real time as reactions proceed within crystals at room temperature (Neutze & Moffat, 2012 ▸). The development of serial femtosecond crystallography (SFX) using X-ray free-electron laser (XFEL)-generated radiation (Chapman *et al.*, 2011 ▸) has greatly enhanced the potential of the field of time-resolved diffraction, since SFX allows (nearly) radiation damage-free data to be collected at room temperature with a time resolution down to the femtosecond range. The first demonstration of time-resolved SFX (TR-SFX) was a major advance for the field since it established that changes in electron density could reliably be extracted from data sets merged from thousands of randomly oriented microcrystals (Tenboer *et al.*, 2014 ▸). This advance allowed the field of time-resolved crystallography to expand (Brändén & Neutze, 2021 ▸) and structural results have been presented from many light-sensitive enzymes, including bacteriorhodopsin (Nango *et al.*, 2016 ▸; Nogly *et al.*, 2018 ▸), photoactive yellow protein (Tenboer *et al.*, 2014 ▸; Pande *et al.*, 2016 ▸), photosystem II (Suga *et al.*, 2017 ▸, 2019 ▸; Kern *et al.*, 2018 ▸; Bhowmick *et al.*, 2023 ▸) and a photosynthetic reaction centre (Dods *et al.*, 2021 ▸). For the field to grow, however, it is essential that time-resolved approaches be applied to the elucidation of structural changes during enzymatic reactions that are not naturally light-sensitive.

Here, we present a room-temperature serial synchrotron crystallography (SSX) structure of SpRub in anticipation of future time-resolved serial crystallography studies of the RuBisCO-catalysed carboxylation of RuBP. SSX data were recorded from microcrystals and yielded an X-ray structure to 2.3 Å resolution, showing excellent electron density of the labile carbamate form of Lys201L in all four molecules of the asymmetric unit.

## 2. Materials and methods

### 2.1. Macromolecule production

**Figure 1:**
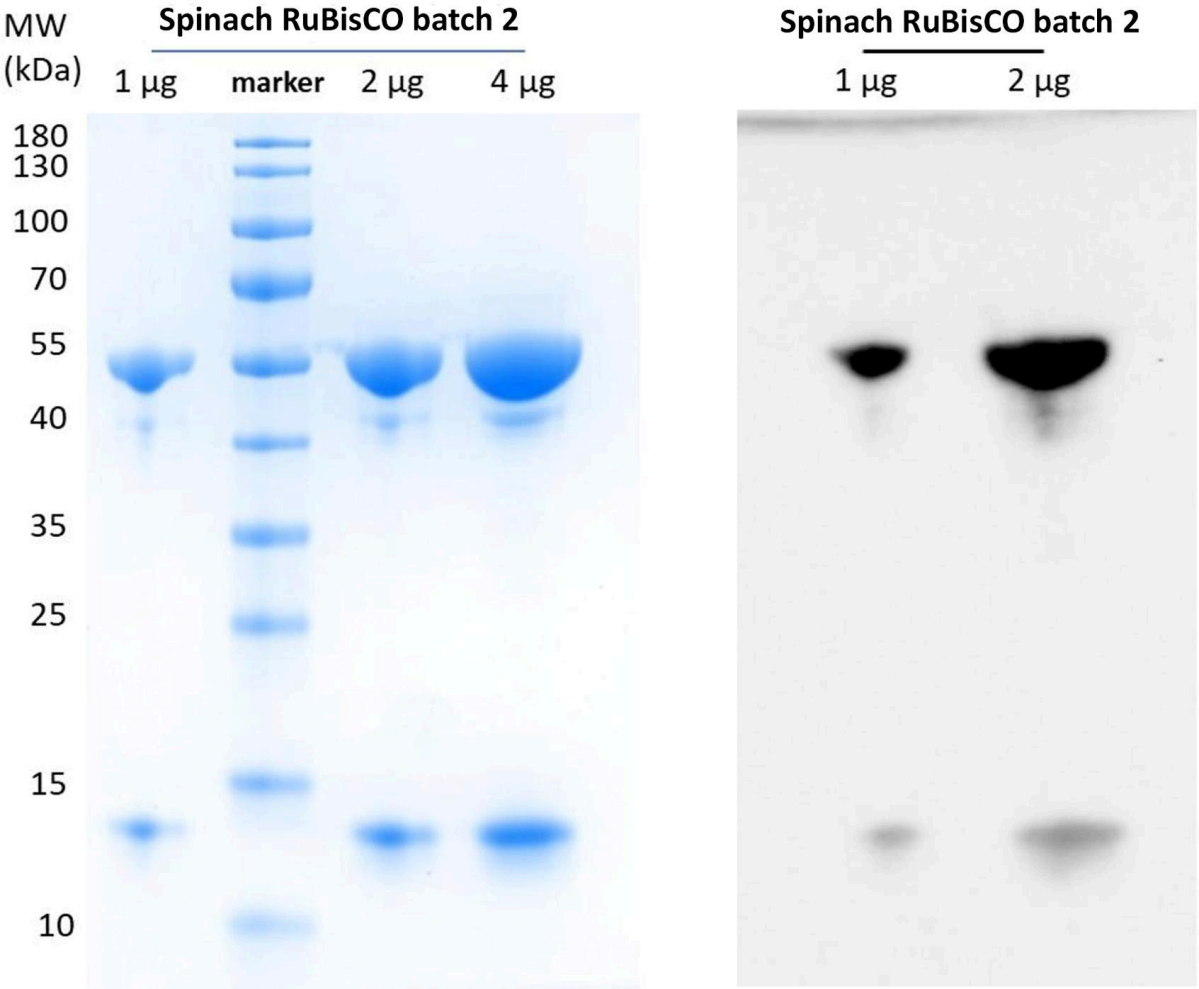
1, 2 and 4 µg of spinach RuBisCO from batch 2 were analysed on an SDS–PAGE gel (left) and 1 and 2 µg were also analysed by Western blot using an anti-RuBisCO antibody. The purity is estimated to be >95%.

SpRub was purified from homogenized fresh spinach leaves (ICA Spenat, ICA Kvantum Malmborgs Clemenstorget, Lund, Sweden) with adjustments to the previously described process (Andersson *et al.*, 1983 ▸). After the preparation of a soluble extract using filtration followed by centrifugation, ammonium sulfate precipitation was performed. The 30–50% fraction was dissolved in 5 m*M* KH_2_PO_4_, 0.1 m*M* EDTA, 1 m*M* DTT pH 7.6 (IEX start buffer) and then dialyzed into the same buffer before loading it onto a HiLoad 26/10 HP Q column (GE Healthcare) for purification by anion-exchange chromatography. Elution was performed using a gradient of 5–250 m*M* KH_2_PO_4_ in 0.1 m*M* EDTA, 1 m*M* DTT pH 7.6; the SpRub peak eluted after the 145 m*M* KH_2_PO_4_ step. After analysis by SDS–PAGE (Fig. 1 ▸) the SpRub-containing fractions were pooled, concentrated and then further purified by gel filtration using a HiPrep 26/60 Sephacryl S-300 HR column (GE Healthcare) in 20 m*M* HEPES, 5 m*M* MgCl_2_ pH 8. Finally, the protein was concentrated to 65 mg ml^−1^ (Wishnick & Lane, 1971 ▸) and snap-frozen in liquid nitrogen.

### 2.2. Crystallization

**Figure 2:**
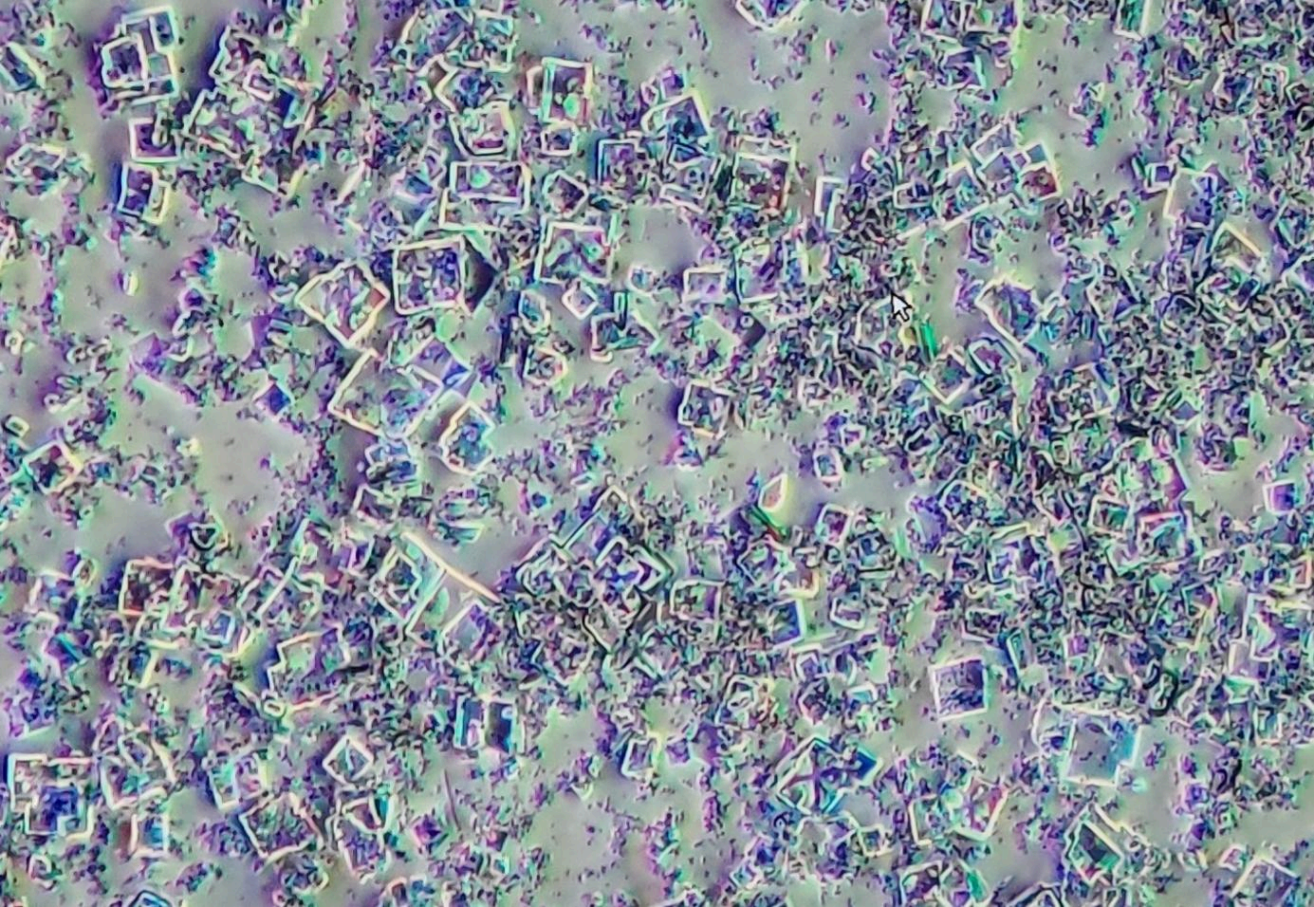
Photograph of the microcrystals of SpRub of 15–40 µm in size that were used in SSX studies.

Due to the absence of reported structures of SpRub obtained using microcrystals, our approach involved initial screening experiments. Two screens, namely JCSG+ and PACT premier (Molecular Dimensions, UK), were used in 96-well plates, with each well containing three drops at different protein:buffer ratios (1:1, 2:1 and 1:2). Promising conditions identified during the screening phase were reproduced and further optimized in 48-well plates for optimization and crystal harvesting. Through multiple optimization iterations of crystallization and data collection, we successfully obtained four conditions that produced large, well diffracting single crystals. These conditions were selected for further optimization in order to obtain microcrystals. Ultimately, microcrystals of SpRub were successfully obtained by the sitting-drop method utilizing an HR1-002 plate (Hampton Research) at a temperature of 20°C (Fig. 2 ▸). The crystallization process involved the activation of SpRub prior to setup (Lundqvist & Schneider, 1991 ▸): a solution of 20 m*M* HEPES pH 8, 50 m*M* MgCl_2_, 50 m*M* NaHCO_3_ was heated to 40° for 30 min and then mixed with the RuBisCO solution and heated at 30° for a further 30 min. The specific crystallization conditions used were as follows: 5 µl SpRub solution at a concentration of 15 mg ml^−1^ (in a buffer consisting of 20 m*M* HEPES, 5 m*M* MgCl_2_ pH 8.0) was combined with 5 µl reservoir solution [0.2 *M* MgCl_2_, 0.1 *M* Tris pH 7.0, 12%(*w*/*v*) PEG 8000]; the total well volume was 500 µl (Table 1 ▸).

### 2.3. SSX data collection

SpRub SSX data were collected at the T-REXX endstation of the P14 beamline operated by EMBL at PETRA-III, DESY, Hamburg, Germany. The protein crystals were loaded onto a silicon chip (Mehrabi *et al.*, 2020 ▸), which was then scanned at room temperature across the X-ray beam (12.7 keV, 10 µm diameter, 1.2 × 10^12^ photons s^−1^) in a HARE pattern (Schulz *et al.*, 2018 ▸) at a rate of 30 positions per second in an attempt to collect time-resolved data. A pulsed 5 ns laser (355 nm) was used to release CO_2_ from a caged compound (Lommel *et al.*, 2013 ▸), with 1 µJ per pulse measured at the sample position (focus diameter 30 µm FWHM). Diffraction images were recorded by an EIGER 4M detector (DECTRIS, Baden-Daettwil, Switzerland) with an exposure time of 5 ms per frame.

### 2.4. Data processing, model building and refinement

Diffraction data were indexed, integrated, merged and converted to MTZ format using *CrystFEL* 0.10.1 (White *et al.*, 2016 ▸; White, 2019 ▸). The indexing rate was 32.1%. Data truncation, phasing and structure refinement were performed in *CCP*4 *Cloud* (Krissinel *et al.*, 2022 ▸) which is part of the *CCP*4 suite (Agirre *et al.*, 2023 ▸). The high-resolution structure of active SpRub (PDB entry 1aus; Taylor & Andersson, 1997*a* ▸) was used as model for molecular replacement with *Phaser* (McCoy *et al.*, 2007 ▸), while an updated sequence from PDB entry 1ir1 (Mizohata *et al.*, 2002 ▸) was used for further model building. The structure was refined by one round of rigid-body refinement using *REFMAC*5 (Nicholls *et al.*, 2018 ▸; Murshudov *et al.*, 1997 ▸, 2011 ▸), followed by several rounds of restrained refinement. Model building was performed in *Coot* (Krissinel & Henrick, 2004 ▸; Emsley *et al.*, 2010 ▸) and all structural representations were generated in *PyMOL* (Schrödinger). A room-temperature SSX structure of active SpRub was obtained at 2.3 Å resolution. This cutoff was decided on because *I*/σ(*I*), CC_1/2_ and *R*_split_ drastically fall and the electron-density maps were of worse quality at resolutions higher than 2.3 Å. Data-collection and refinement statistics are presented in Tables 2 ▸ and 3 ▸, respectively.

## 3. Results and discussion

SpRub microcrystals were grown from purified protein after iterations of screening, optimization to obtain larger crystals and final optimization that yielded homogeneous microcrystals that were initially tested at BioMAX (Ursby *et al.*, 2020 ▸). The microcrystals were mounted on a silicon chip and SSX data were collected at the T-REXX experimental setup of beamline P14 of PETRA III (Mehrabi *et al.*, 2020 ▸) in an attempt at TR-SSX of a CO_2_-caged compound (Lommel *et al.*, 2013 ▸). Serial crystallography data were collected from seven separate chips. The TR-SSX attempt was not successful, but fully complete X-ray diffraction data with a multiplicity of 770 were collected to 2.3 Å resolution (Table 2 ▸). As with earlier models, SpRub crystallized in space group *C*222_1_ with four copies of the RbcL and RbcS subunits present in the asymmetric unit, such that two symmetry-related copies of the asymmetric unit form the biologically functional hexadecameric oligomer. Molecular replacement successfully used an earlier structure of SpRub (PDB entry 1aus) as a search model, and structural refinement yielded a model with *R* and *R*_free_ factors of 18.2% and 22.8%, respectively. No density for released CO_2_ could be detected.

### 3.1. Structural comparisons on C^α^ atoms

**Figure 3:**
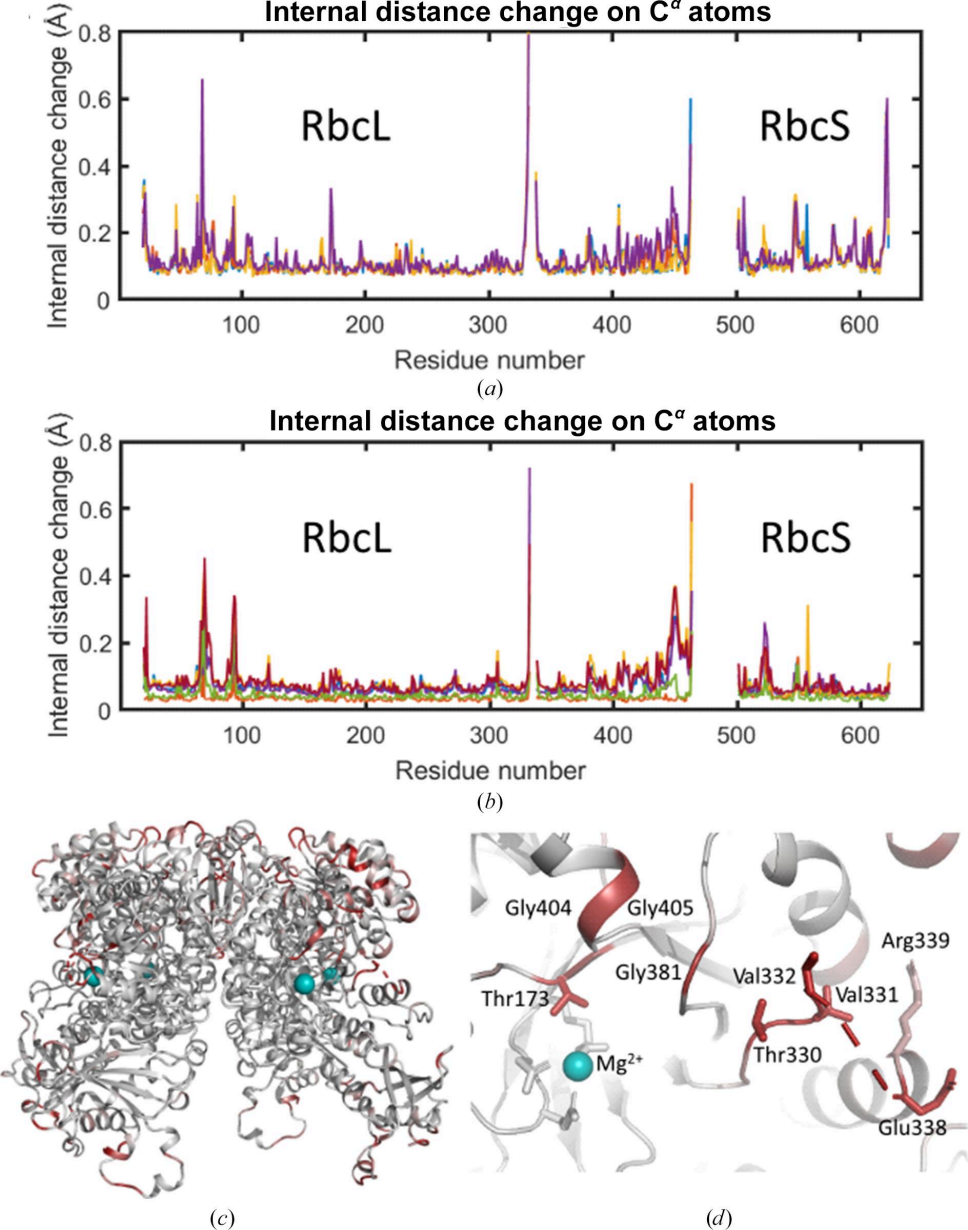
Structural comparisons of C^α^-atom coordinates of the SSX structure of SpRub with an earlier single-crystal structure (PDB entry 1aus) or between protomers. (*a*) Average internal distance matrix displacements of C^α^ atoms when compared with the coordinates of PDB entry 1aus. These plots are shown for all four copies of the large (RbcL) and small (RbcS) RuBisCO subunits within the asymmetric unit. (*b*) Average internal distance matrix displacements of C^α^ atoms when compared with the coordinates of other copies of the large (RbcL) and small (RbcS) RuBisCO subunits within the asymmetric unit. Since there are four copies, six comparisons are plotted in total. This plot highlights the regions which differ most strongly between the subunits. (*c*) Colour representation of the mean displacements of C^α^ atoms relative to PDB entry 1aus, where white represents no displacement and dark red represents the maximum displacement. Most regions in which significant structural changes are observed are on the surface of the protein. (*d*) The same representation as used in (*c*) but illustrating structural changes near the active site of the protein. No significant displacements of active-site residues are observed, only the loose region of glycines.

Overall, the room-temperature SSX structure of SpRub and that in PDB entry 1aus (Taylor & Andersson, 1997*a* ▸) are very similar. An internal distance matrix analysis of C^α^-atom positions was used to compare the structures (Schneider, 2000 ▸; Wickstrand *et al.*, 2015 ▸) since this allows the structures to be compared without the need to align them. This analysis showed that the internal distances between C^α^ atoms between PDB entry 1aus and the SSX structure differ by only 0.11 Å when averaged over the four copies of RbcL and by 0.12 Å when averaged over the four copies of RbcS (Fig. 3 ▸*a*). These values are slightly higher than the corresponding average internal distance changes between the four copies of the molecule within the asymmetric unit, with the mean difference between the four protomers being 0.07 Å for RbcL and 0.06 Å for RbcS (Fig. 3 ▸*b*).

By mapping the average changes in internal distances on C^α^ atoms (Fig. 3 ▸*a*) onto the protein structure (Fig. 3 ▸*c*), we observe that several of the regions that show the largest structural perturbations relative to PDB entry 1aus are located on the surface of the protein. Close to the active site of the protein, we note that Thr173L as well as three glycine residues (Gly381L, Gly404L and Gly405L) show small but significant displacements relative to their positions in PDB entry 1aus. Since glycine residues introduce additional flexibility into the allowed backbone conformations, it is perhaps unsurprising that glycine-rich regions show modest disparities. From a functional perspective, it is significant that none of the residues that coordinate the active-site Mg^2+^ show significant displacements.

### 3.2. Electron density for the active site of the protein

**Figure 4:**
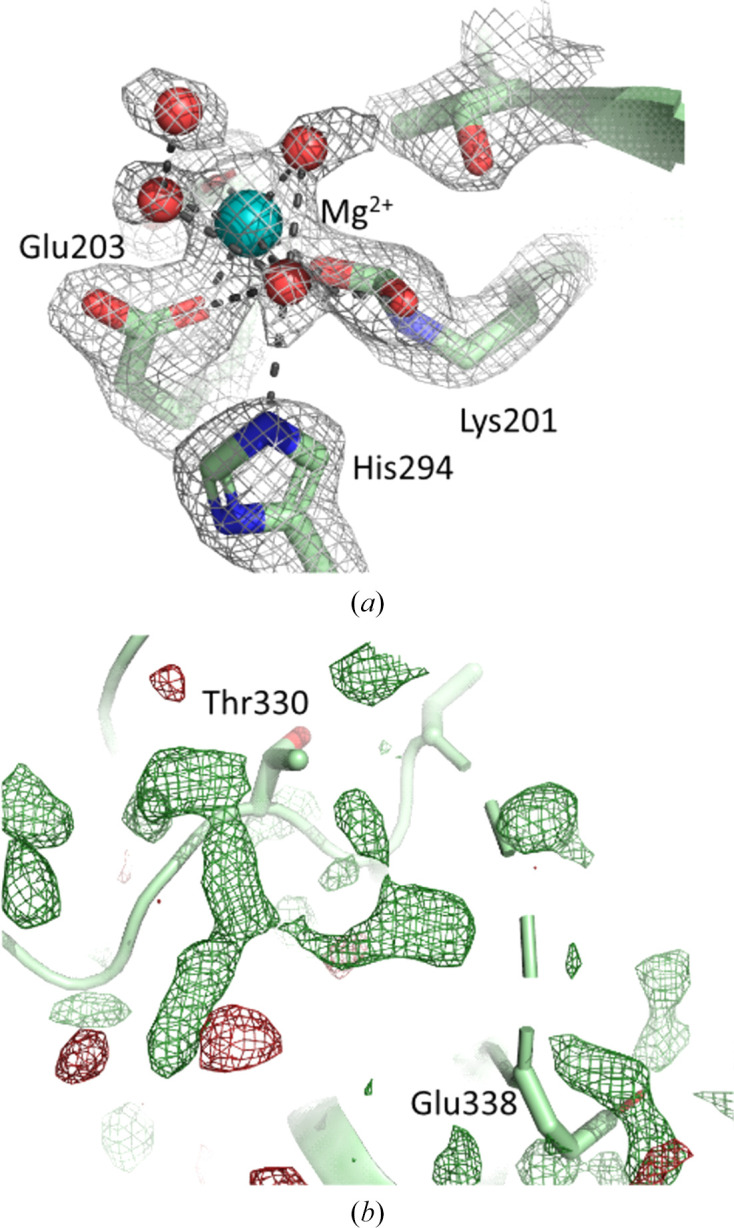
Electron density near the active site of SpRub. (*a*) 2*F*_obs_ − *F*_calc_ electron-density map (grey) showing the quality of the map at the active site of the protein. The labile carbamate form of Lys201L is clearly visible and has continuous electron density between the covalently bound CO_2_ and the lysine side chain. The active-site Mg^2+^ ion shows identical ligands and water-molecule coordination to earlier structures of RuBisCO, but with an additional ordered water molecule on the outskirts of this water cluster (the density is contoured at 1.2σ). (*b*) *F*_obs_ − *F*_calc_ residual electron-density map (forest green) showing residual, semi-continuous electron density near the disordered region from residues 333L to 337L. However, it was not possible to model this section into this density and there may be multiple conformations that overlap with each other in this region (the density is contoured at 2.5σ).

All four RbcL protomers in the SSX structure show well ordered electron density at the active site of the protein. Specifically, continuous electron density covalently connects the CO_2_ molecule to Lys201L, and the labile carbamate form of this residue coordinates Mg^2+^ (Fig. 4 ▸*a*). Two other negatively charged residues, Asp203L and Glu204L, coordinate Mg^2+^, as previously observed in the activated structure of RuBisCO without any ligand or substrate (Taylor & Andersson, 1997*a* ▸). In addition, three water molecules coordinate Mg^2+^ to give the cation its characteristic sixfold octahedral coordination geometry. A fourth water molecule is well ordered in the immediate vicinity of the active site, coordinating one water molecule that is bound to Mg^2+^. The earlier single-crystal structure of the activated state of RuBisCO solved at 2.2 Å resolution (PDB entry 1aus) shows three water molecules coordinating Mg^2+^ in one of the four protomers of RbcL, but these water ligands were not built in all four protomers. Nevertheless, residual *F*_obs_ − *F*_calc_ electron density in PDB entry 1aus suggests that they could also have been modelled in all protomers. A higher 1.6 Å resolution single-crystal structure, but with bound inhibitor (Taylor *et al.*, 1996 ▸), reveals that the presence of a transition-state analogue (2-carboxy­arabinitol bisphosphate) causes all three ligating water molecules to be displaced, with Mg^2+^ becoming coordinated by the C2 hydroxyl, the C3 hydroxyl and the 2′-carboxyl atoms of 2-carboxyarabinitol bisphosphate. Irrespectively, these observations suggest that the SSX active-structure arrangement is consistent with previous work at similar resolution.

A short loop of residues, 333L–337L, lacked the electron density to be modelled in our structure. Significant *F*_obs_ − *F*_calc_ residual electron density is visible in this region (Fig. 4 ▸*b*) yet, while continuous to some extent, it is not possible to easily model the four missing residues within this residual density. Therefore, this short missing section was not included within our model. It seems possible that there will be more than one conformation in this region. Moreover, structural predictions using *AlphaFold* (https://alphafold.ebi.ac.uk/entry/P00875; Jumper *et al.*, 2021 ▸) also show this region to be disordered.

### 3.3. Time-resolved SSX data-collection attempt

In addition to a reaction-initiation protocol, a time-resolved X-ray diffraction study of enzymatic turnover requires either that the timescale of substrate binding to the protein in microcrystals is at least as rapid as the desired time resolution of the experiment or that the substrate is bound in the microcrystals in an inert form that can be activated by another means. In this work, we envisioned the use of UV-light release of photocaged CO_2_ as a means of initiating the enzymatic reaction. We therefore attempted to find conditions in which a stable complex of SpRub and RuBP could be obtained. Previous studies using single-crystal diffraction successfully determined an X-ray structure of the SpRub–RuBP complex to 2.1 Å resolution with the activated carbamate form of Lys201L, but with Mg^2+^ substituted with Ca^2+^ so as to trap the substrate as a stable complex (Taylor & Andersson, 1997*b* ▸). Moreover, a slightly lower resolution complex structure was also determined at 2.4 Å resolution but without Lys201L being activated.

In our studies, when RuBP was added to SpRub microcrystals they rapidly dissolved, and this phenomenon could clearly be observed in real time under a microscope. It is possible that this sensitivity to substrate is due to enzymatic activity and reflects the presence of either CO_2_ or O_2_ substrate within our microcrystal preparations. Nevertheless, the sensitivity of these microcrystals to substrate was reduced when they were immersed in monoolein to generate a semi-viscous gel for sample injection. In this manner, it was possible to determine an SSX structure of the enzyme–substrate complex but only to 3.8 Å resolution (unpublished data) and not under conditions suitable for time-resolved experiment using a caged compound.

## 4. Conclusion

Time-resolved serial X-ray crystallography is a rapidly growing field of research that has encountered many challenges in sample preparation and reaction initiation (Brändén & Neutze, 2021 ▸). RuBisCO is a potential target for such experiments since there is tremendous scientific interest in the chemical details of how CO_2_ is removed from the atmosphere and incorporated into the biosphere on both agricultural and environmental grounds. Moreover, as a relatively slow reaction (with a turnover of the order of 100 ms), the timescale is well suited to validation using synchrotron radiation, even if TR-SFX studies using XFEL radiation may ultimately be necessary to optimize the resolution of the collected data and to minimize the effects of X-ray-induced photoreduction of metal centres.

Here, we explored the foundations for a potential future TR-SSX study of SpRub. Most importantly, we obtained a room-temperature SSX structure of SpRub at 2.3 Å resolution, which showed high agreement with earlier single-crystal structures of this enzyme without any obvious signs of radiation damage. As such, our data show that high-quality structures can be determined using serial crystallography protocols at synchrotron-radiation sources. Since the microcrystals were grown using protein purified from a highly abundant native source, sample quantities will not be a limiting factor. However, initial attempts to soak substrate into microcrystals either failed or led to the crystals dissolving and the quality of the X-ray diffraction data being severely compromised. As such, this strategy will need to be optimized, potentially by taking more care to minimize any potential sources of CO_2_ or O_2_ before the crystals are mixed with the starting sugar, testing whether viable strategies exist for co-crystallization of enzyme and substrate and even exploring new crystallization conditions.

The atomic coordinates and structure-factor files for the room-temperature SSX SpRub data have been deposited in the Protein Data Bank (https://www.pdb.org) as PDB entry 8qj0.

## Supplementary Material

PDB reference: room-temperature serial synchrotron crystallography structure of *Spinacia oleracea* RuBisCO, 8qj0

## Figures and Tables

**Table 1:** Crystallization

Method	Vapour diffusion, sitting drop
Plate type	HR1-002 plate, Hampton Research
Temperature (K)	293
Protein concentration (mg ml^−1^)	15
Buffer composition of protein solution	20 m*M* HEPES, 5 m*M* MgCl_2_ pH 8.0
Composition of reservoir solution	0.2 *M* MgCl_2_, 0.1 *M* Tris pH 7.0, 12%(*w*/*v*) PEG 8000
Volume and ratio of drop	10 µl, 1:1 ratio
Volume of reservoir (µl)	500

**Table 2:** Data collection and processing Values in parentheses are for the highest resolution shell.

Diffraction source	T-REXX, PETRA III
Wavelength (Å)	0.976
Temperature (K)	294
Detector	EIGER 4M
Crystal-to-detector distance (mm)	120.5
Exposure time per image (s)	0.005
Space group	*C*222_1_
*a*, *b*, *c* (Å)	158.60, 157.12, 202.74
α, β, γ (°)	90, 90, 90
Resolution range (Å)	97.78–2.30 (2.38–2.30)
Total No. of reflections	86314652
No. of unique reflections	112061
Completeness (%)	100 (100)
Multiplicity	770 (528)
〈*I*/σ(*I*)〉	7.96 (3.66)
CC_1/2_	0.9943 (0.9523)
*R*_split_† (%)	9.52 (23.88)
Overall *B* factor from Wilson plot (Å^2^)	20.24

† *R*_split_ = 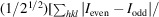

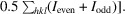

**Table 3:** Structure solution and refinement Values in parentheses are for the highest resolution shell.

Resolution range (Å)	97.78–2.30 (2.382–2.300)
Completeness (%)	100 (100)
No. of reflections, working set	111773 (11108)
No. of reflections, test set	5435 (564)
Final *R*_work_ (%)	18.11
Final *R*_free_ (%)	22.74
No. of non-H atoms	
Protein	17861
Ligand	16
Water	987
Total	18864
R.m.s. deviations	
Bond lengths (Å)	0.015
Angles (°)	2.16
Average *B* factors (Å^2^)	
Protein	25.17
Ligand	19.09
Water	25.05
Ramachandran plot statistics	
Most favoured (%)	96
Allowed (%)	2.92
